# The interaction between vaginal microbiota, cervical length, and vaginal progesterone treatment for preterm birth risk

**DOI:** 10.1186/s40168-016-0223-9

**Published:** 2017-01-19

**Authors:** Lindsay M. Kindinger, Phillip R. Bennett, Yun S Lee, Julian R. Marchesi, Ann Smith, Stefano Cacciatore, Elaine Holmes, Jeremy K. Nicholson, T. G. Teoh, David A. MacIntyre

**Affiliations:** 1 Imperial College Parturition Research Group, Division of the Institute of Reproductive and Developmental Biology, Department of Surgery and Cancer, Faculty of Medicine, Imperial College London, Hammersmith Campus, London, W12 0NN UK; 2 Queen Charlotte’s Hospital, Imperial College Healthcare NHS Trust, London, UK; 3 Department of Obstetrics and Gynaecology, St Mary’s Hospital, Imperial College Healthcare NHS Trust, London, UK; 4 Centre for Digestive and Gut Health, Department of Surgery and Cancer and the Institute of Global Health Innovation, Faculty of Medicine, Imperial College London, London, UK; 5 School of Biosciences, Cardiff University, Cardiff, UK; 6 Division of Computational Systems Medicine, Department of Surgery and Cancer, Faculty of Medicine, Imperial College London, London, UK

**Keywords:** Vaginal microbiome, Progesterone, Lactobacillus, Preterm birth, Cervical length

## Abstract

**Background:**

Preterm birth is the primary cause of infant death worldwide. A short cervix in the second trimester of pregnancy is a risk factor for preterm birth. In specific patient cohorts, vaginal progesterone reduces this risk. Using 16S rRNA gene sequencing, we undertook a prospective study in women at risk of preterm birth (*n* = 161) to assess (1) the relationship between vaginal microbiota and cervical length in the second trimester and preterm birth risk and (2) the impact of vaginal progesterone on vaginal bacterial communities in women with a short cervix.

**Results:**

*Lactobacillus iners* dominance at 16 weeks of gestation was significantly associated with both a short cervix <25 mm (*n* = 15, *P <* 0.05) and preterm birth <34^+0^ weeks (*n* = 18; *P <* 0.01; 69% PPV). In contrast, *Lactobacillus crispatus* dominance was highly predictive of term birth (*n* = 127, 98% PPV). Cervical shortening and preterm birth were not associated with vaginal dysbiosis. A longitudinal characterization of vaginal microbiota (<18, 22, 28, and 34 weeks) was then undertaken in women receiving vaginal progesterone (400 mg/OD, *n* = 25) versus controls (*n* = 42). Progesterone did not alter vaginal bacterial community structure nor reduce *L. iners*-associated preterm birth (<34 weeks).

**Conclusions:**

*L. iners* dominance of the vaginal microbiota at 16 weeks of gestation is a risk factor for preterm birth, whereas *L. crispatus* dominance is protective against preterm birth. Vaginal progesterone does not appear to impact the pregnancy vaginal microbiota. Patients and clinicians who may be concerned about “infection risk” associated with the use of a vaginal pessary during high-risk pregnancy can be reassured.

**Electronic supplementary material:**

The online version of this article (doi:10.1186/s40168-016-0223-9) contains supplementary material, which is available to authorized users.

## Background

Preterm birth before 37 weeks of gestation is now the leading cause of death among children under the age of five [1]. An estimated 15 million babies are born preterm each year, and in the USA alone, the annual healthcare costs associated with those babies that survive is in excess of $25 billion [2]. Ascending bacterial infection from the vagina through the cervix into the uterine cavity is considered to be a major cause of spontaneous preterm birth [3–5]. Maternal host-vaginal microbial interactions throughout pregnancy are likely to play a fundamental role in reproductive health outcomes. Unlike other body sites where high bacterial diversity is considered beneficial to health [6, 7], a healthy vaginal community structure in pregnancy is dominated by only one, or a few, *Lactobacillus* species [8, 9]. These species provide protection against pathobiont colonization through excretion of lactic acid and production of antimicrobial compounds [10]. Assessment of vaginal microbial community structure can be performed using a variety of next generation sequencing and PCR-based platforms. The resulting data is typically analyzed using multivariate clustering approaches that permit comparison of individual taxa or community compositions [11–13]. A commonly used classification scheme involves hierarchical clustering analysis of 16S rRNA gene sequencing data into community state types (CSTs) as first described by Ravel and colleagues [13]. CSTs are typically dominated by one of four *Lactobacillus* species; *Lactobacillus crispatus* (CST I), *Lactobacillus gasseri* (CST II), *Lactobacillus iners* (CST III), and *Lactobacillus jensenii* (CST V). CST IV describes microbial communities largely devoid of *Lactobacillus* species and enriched mainly in anaerobic bacteria (CST IV).

The composition of vaginal CSTs appears to be influenced by endogenous hormones, fluctuating with menses [14], the use of oral contraceptives [15] and onset of menopause [16], and estrogen supplementation in post-menopausal states [17]. In pregnancy, elevated concentrations of circulating estrogen drive glycogen accumulation in the vaginal epithelium, which is broken down by host α-amylase to complex sugar products such as maltotetraose, maltotriose, and maltose providing carbon sources preferentially utilized by *Lactobacillus* species [18]. This substrate availability leads to increased *Lactobacillus* species abundance and stability with advancing gestation and a decline in the number of women harboring microbial communities void of lactobacilli, with a dramatic change to reduced *Lactobacillus* dominance and increased diversity following estrogen withdrawal in the post-partum period [8, 19]. There is an association between *Lactobacillus* spp. depletion and vaginal dysbiosis and poor pregnancy outcomes including preterm birth [19] and late miscarriage [20], which appears to be patient cohort dependent [9]. Thus, the interplay between hormonal and metabolic signaling at the vaginal mucosa interface may act as a protective mechanism for the immuno-modulated pregnant mother, against exposure to pathogenic bacteria [21]. This may influence not only immediate pregnancy outcomes but also longer immunological health in the neonate such as allergy and asthma [22, 23]. This vaginal dysbiosis is present in between 2 and 27% of the population in pregnancy but does not always appear to be pathogenic [8, 24]. Recent evidence implicates *L. iners* dominance rather than dysbiosis for preterm birth risk in pregnancy [25]. *L. iners* is a vaginal commensal that has relatively recently been shown to be associated with dysbiosis [26, 27] and has been suggested as marker of microbial imbalance leading to BV [28].

The cervix serves as both a mechanical and chemical barrier to ascending bacteria [29]. Premature cervical ripening, a prerequisite for the expulsion of the fetus, may be triggered by exposure of the amniotic cavity to pathogenic bacteria ascending from the vagina [30], which drives a pro-inflammatory cytokine response, triggering prostaglandin release, and untimely cervical remodeling, softening, and dilation [31]. Premature cervical ripening is detectable by transvaginal ultrasound (TVS) several weeks prior to the onset of the clinical symptoms of preterm labor. Second trimester transvaginal cervical length measurements are considered a reliable and predictive tool for preterm birth and are frequently used for preterm birth surveillance [32]. Pregnant women with a short cervix, <25 mm before 24 weeks of gestation are considered at highest risk of preterm birth [33]. Early diagnosis of these pregnancies enables timely and targeted intervention by either cervical cerclage or vaginal progesterone therapy [34]. While both prevention strategies display comparable efficacy [35], progesterone supplementation is increasingly used as it negates the surgical risks associated with cerclage insertion such as maternal pyrexia, vaginal infection, bleeding, and subsequent requirement for cesarean section [36, 37] and has not been associated with any adverse neonatal effects [38, 39].

The mechanism of action of vaginal progesterone in the prevention of preterm birth involves its capacity to promote anti-inflammatory and pro-relaxant pathways in the uterus thereby reducing uterine contractility [40–43]. Endogenous progesterone is thought to largely exert anti-inflammatory downstream effects via progesterone receptor B-dominant signaling [43] to inhibit nitric oxide, prostaglandin, and cytokine production [41, 42]. This ultimately reduces myometrial contractility thereby inhibiting premature onset of labor [40]. Progesterone also exerts a quiescent effect on the cervix by limiting prostaglandin-induced collagenous remodeling of the cervical fibroblast [44–46]. Progesterone supplementation is therefore prescribed primarily for its systemic anti-inflammatory actions to maintain myometrial and cervical quiescence in high-risk pregnancies. In clinical studies, vaginal progesterone has been shown to attenuate the rate of cervical shortening [47, 48], but its efficacy in preterm birth prevention is cervical-length dependent [48–51] with most benefit observed in high-risk pregnancies with a short cervix <25 mm [35]. Administration of vaginal progesterone to women with a long cervix has not been shown to improve pregnancy outcome [51]. Studies on the impact of progesterone on the composition of the vagina microbiota are limited. In non-pregnant women, Borgdorff et al. [52] found that both injectable progestin contraception and combined oral contraception (progestin and estrogen) do not significantly alter vaginal microbiota, but may increase the risk of HIV transmission [53]. It is has been hypothesized that this susceptibility relates to a progesterone-induced modulation of the local inflammatory immune response to infection or alternately thinning of the vaginal epithelial barrier [54]. The impact of vaginal progesterone pessaries on the composition of vaginal microbial communities in pregnancies at risk of preterm birth is unknown.

We hypothesized that progesterone supplementation would promote *Lactobacillus* spp. dominance and stability, given progesterone’s anti-inflammatory properties, and efficacy for preterm birth prevention. We therefore undertook a prospective study in women at high-risk of preterm birth to assess (1) the relationship between vaginal microbiota in the second trimester and the risk of preterm birth and (2) the impact of vaginal progesterone therapy on the vaginal microbiota in women with a short cervix.

## Methods

The study was approved by NHS National Research Ethics Service (NRES) Committee London - City and East (REC 12/LO/2003), and all participants provided written, informed consent at enrolment. A workflow of the study is provided in Additional file 1.

### Patient details and sample collection for cross-sectional recruitment

To investigate the association between the vaginal microbiome, cervical length, and preterm birth outcome, a cross-sectional cohort of singleton pregnancies at higher than normal risk of preterm birth (due to a history of previous spontaneous preterm birth <37 weeks^+0^ ^days^) were prospectively recruited from the preterm surveillance clinics at two tertiary London maternity units between January 2013 and August 2014. At initial attendance of preterm surveillance clinics at 16 weeks of gestation, cervico-vaginal fluid was sampled from the posterior fornix under direct visualization, using a BBL™ CultureSwab™ MaxV Liquid Amies swab (Becton, Dickinson and Company, Oxford, UK). The vaginal swabs were placed immediately on ice before being transferred and stored at −80 °C within 5 min of collection. A cervical length (CL) measurement was taken by transvaginal scan (TVS) in supine position, with an empty bladder, taking care to avoid undue pressure on the cervix. Metadata collected included gestation age at sampling, subsequent interventions for preterm birth, gestation at birth, BMI, ethnicity, and antibiotics within the week preceding sampling. Participation in this study did not influence subsequent clinical care or dictate preventative interventions (cervical cerclage or vaginal progesterone supplementation) for preterm birth risk. For the duration of the study, both units employed a policy of CL screening every 3 weeks until 25 weeks, with the indication for intervention being a CL <25 mm at TVS measured at ≤23^+6^ weeks gestation. In this cross-sectional arm of the study, the choice of intervention for a short cervix (cerclage or progesterone) was at the discretion of the attending clinician.

### Longitudinal sampling following progesterone intervention

A longitudinal study was undertaken in a new pregnancy cohort to assess the effect of progesterone therapy on pregnancy with a short cervix. Women with a prior preterm birth <37 weeks were prospectively recruited from the same preterm surveillance clinics. At initial screening ≤18 weeks, all women underwent vaginal swab sampling for 16S rRNA gene sequencing, followed by CL measurement at TVS, as previously described. Based on CL measurement, women were then allocated into one of two groups. Women with a short cervix <25 mm were treated with vaginal progesterone 400 mg OD at night to continue until 34 completed weeks gestation. Women with a CL ≥25 mm were used as controls and did not receive progesterone or cerclage for the remainder of their pregnancy. Both progesterone and control groups were recruited at ≤18 weeks, and vaginal swab samples were collected longitudinally at 22, 28, and 34 weeks of gestation.

Eligibility criteria for participation in both cross-sectional and longitudinal studies included women with a singleton pregnancy and a prior spontaneous preterm birth <37 weeks^+0 days^, who had not undergone CL screening or received either progesterone or cerclage intervention prior to recruitment. Exclusion criteria included multiple pregnancy, preterm pre-labor rupture of membranes (PPROM), iatrogenic preterm birth, HIV positive women, and women who had had sexual intercourse or vaginal bleeding in the preceding 48 h. In the longitudinal study, any women receiving cervical cerclage in either the progesterone or control groups were excluded as this may adversely impact on vaginal microbiota [37].

### DNA extraction and 16S rRNA gene sequencing

DNA extraction from BBL™ CultureSwab™ was performed as previously described [8]. Forward and reverse fusion primers were used to amplify the V1-V3 hypervariable regions of 16S rRNA genes. The forward primer was made up of an Illumina i5 adapter (5′-3′) (AATGATACGGCGACCACCGAGATCTACAC), 8 bp barcode, primer pad (forward: TATGGTAATT), and the 28F-GAGTTTGATCNTGGCTCAG primer [55]. The reverse fusion primer consisted of an (5′-3′) Illumina i7 adapter (CAAGCAGAAGACGGCATACGAGAT), 8 bp barcode, primer pad (reverse: AGTCAGTCAG), and the reverse primer (519R-GTNTTACNGCGGCKGCTG). Sequencing was performed on an Illumina MiSeq platform (Illumina, Inc. San Diego, California). Sequence data was processed and analyzed using the MiSeq SOP Pipeline of the Mothur package [56] with the Silva bacterial database (www.arb-silva.de/) used for sequence alignment. Sequence classification was performed using the RDP database reference sequence files and the Wang method [57] and taxonomy assignments determined using the RDP MultiClassifier script and USEARCH with 16S rRNA gene sequences from the cultured representatives from the RDP database [58] for species level taxonomies. Data was re-sampled and normalized to the lowest read count in Mothur (*n* = 725) [59].

### Statistical analyses

Examination of statistical differences between vaginal microbiota was performed using the Statistical Analysis of Metagenomic Profiles (STAMP) software package [60]. To classify vaginal bacterial communities into community state types (CSTs), hierarchical clustering analysis (HCA) species taxonomy was performed using ward linkage with a clustering density threshold of 0.75. Samples were classified into five CSTs; I (*L. crispatus*), II (*L. gasseri*), III (*L. iners*), IV (mixed bacterial species), and V (*L. jensenii*) as described by Ravel et al. [13].

#### Cross-sectional cohort

In the cross-sectional cohort, a total number of species observed and the Shannon index of alpha diversity were calculated and compared across gestation at birth: <34^+0^ weeks, 34^+0^ to 36^+6^ weeks, and ≥37^+0^ weeks, using a 2-way ANOVA.

Examination of the relationship between vaginal bacterial communities (or CSTs) and cervical length or preterm birth was assessed using a Fisher exact test as to provide an exact, robust *P* value that is less sensitive to small sample sizes than alternative approaches such as Chi-squared (reference). Fisher’s exact test was also used to examine individual CST assignments (in 5 × 2 contingency tables for cervical length and requirement for future interventions and 3 × 25 contingency tables for birth gestation and ethnicity). Gehan-Breslow-Wilcoxon test was used to compare pregnancy survival (duration of gestation) for CST I compared to CST III [61].

A logistic mixed-effects regression analysis incorporating gestational age at sample, maternal age, and BMI as fixed effects and ethnicity as a random effect was performed to assess the relationship between pregnancy outcome (birth <34 and >34 weeks) and CSTs as well as individual species abundance. Analyses were performed in R using ANOVA and the “glm” (generalized linear model) R function to analyze the table of deviance for CSTs and for individual species (present in >5% of samples). Species abundances were log-transformed, adjusted for confounders, and false discovery rate adjustment (Benjamini-Hochberg) was applied to correct *P* values for each analysis [62].

Accuracy parameters, sensitivity (sens), specificity (spec), positive predictive values (PPV), and negative predictive values (NPV) were calculated for the prediction of preterm birth <34^+0^ weeks according to CST classification at 16-week sampling.

#### Longitudinal cohort

A linear mixed-effects model incorporating gestational age, maternal age, BMI, ethnicity (Asian, Black, or Caucasian), and cohort (progesterone with short cervix versus control with normal cervix) as fixed-effects and the anonymized patient ID as uncorrelated random-effect was used to assess the impact of progesterone intervention on CST distribution and relative abundance in species present in >5% of all samples. The contributions of fixed-effects terms (*P* value and *F* statistics) were calculated using ANOVA with Satterthwaite approximation for degrees of freedom. For each fixed-effects term, a false discovery rate adjustment (Benjamin-Hochberg) was applied to correct *P* values. All data and computational approaches used for this study are provided in additional information (Additional files 2, 3, 4, 5, and 6).

## Results

### Cross-sectional study patient cohort demographics

A total of 161 pregnant women attending prematurity surveillance clinics for their first appointment (mean 16^+6^ weeks gestation, Table 1) consented to a vaginal swab followed by a transvaginal scan for cervical length measurement. Spontaneous preterm birth <37 weeks occurred in 34 women (21%) (mean 32^+6^ weeks, SD ± 3^+6^ weeks, range 24^+4^–36^+6^ weeks). Rates of preterm birth <37^+0^ weeks were higher in Black women (37%, 11/30) than Caucasians (17%, 18/104) and Asians (19%, 5/27; *P <* 0.05). Subsequent cervical shortening to below 25 mm occurred in 66% (91/161), all of whom went on to receive an intervention (ultrasound indicated cervical cerclage, *n* = 71 or vaginal progesterone, *n* = 20).

**Table 1:** Patient demographics for a cross-section of 161 participants

	Term birth >37 weeks	Preterm birth <37 weeks	Total
*n*/*N* (%)	127/161 (79%)	34/161 (21%)	161/161 (100%)
BMI			
Mean ± SD (range)	24.3 ± 4.4 (18–48)	24.3 ± 4.4 (18.4–35)	24.3 ± 4.4 (18–48)
Ethnicity, *n*/*N* (%)			
Caucasian	86/127 (68%)	18/34 (53%)	104/161 (65%)
Asian	22/127 (17%)	5/34 (15%)	27/161 (17%)
Black	19/127 (15%)	11/34 (32%)*	30/161 (19%)
Smoker, *n*/*N* (%)	8/127 (6%)	3/34 (9%)	11/161 (7%)
Gestation at sample (weeks)			
Mean ± SD (range)	17^+0^ ± 1.0 (13^+1^–18^+4^)	16^+4^ ± 1.4 (12^+1^–18^+4^)	16^+6^ ± 1.1 (12^+1^–18^+4^)
Cervical length (mm)			
Mean ± SD (range)	32.5 ± 1.0 (18–50)	30.6 ± 6.4 (10–40)	32 ± 5.6 (10–50)
Intervention *n*/*N* (%)			
No intervention	60/127 (47%)	10/32 (29%)	70/161 (43%)
Cerclage	51/127 (40%)	20/32 (59%)	71/161 (44%)
Progesterone	16/127 (13%)	4/32 (12%)	20/161 (12%)
Gestation at delivery, *n*/*N* (%)			
Early PTB, <34^+0^ weeks	na	18/34 (53%)	18/161 (11%)
Late PTB, 34^+0^ to <37^+0^ weeks	na	16/34 (47%)	16/161 (10%)
Term, ≥37^+0^ weeks	127/127 (100%)	na	127/161 (79%)

*PTB* preterm birth, *na* not applicable

*P < 0.05 Fisher’s exact term vs. preterm birth groups

### The vaginal microbiome at 16 weeks in high-risk pregnancy

Using hierarchical clustering analysis (HCA) of normalized genera taxonomy read counts, vaginal swab samples were classified into three categories; normal (>90% *Lactobacillus* spp., 147/161, 91%), intermediate (50–90% *Lactobacillus* spp., 5/161, 3%), and dysbiotic (<10% *Lactobacillus* spp., 9/161, 6%; Additional file 7A). No relationship was observed between genera level structure and subsequent gestational age at delivery. Dominance of *Lactobacillus* species occurred in equal proportions of patients experiencing preterm <37 weeks (31/34; 91%) or term birth (116/127; 91%). Of those women delivering preterm, 3/34 (9%) harbored a dysbiotic or intermediate microbiome at 16 weeks compared to 6/127 (5%) who delivered at term. Consistent with these findings, measurements of species richness (total number of species observed; Additional file 7B) and alpha diversity (Shannon index; Additional file 7C) at 16 weeks were comparable between women experiencing term (≥37^+0^ weeks, *n* = 127), late preterm (34^+0^–36^+6^ weeks, *n* = 16), and early preterm (<34^+0^ weeks, *n* = 18) delivery.

**Fig. 1:**
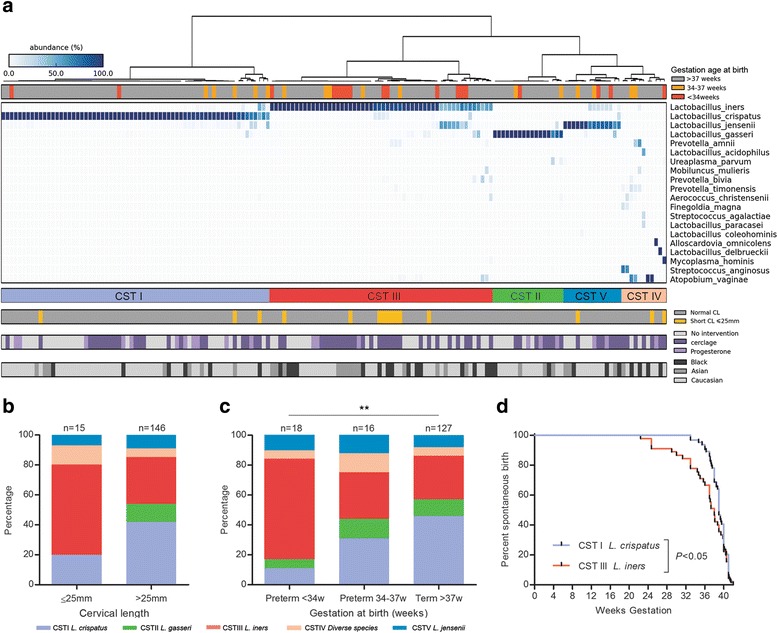
*L. iners* dominance is associated with a short cervix and preterm birth risk while *L. crispatus* is protective. **a** Heatmap of vaginal species data correlated community state types of samples (*n* = 161) with ethnicity, cervical length <25 mm, subsequent cerclage or progesterone intervention, and gestation at birth. **b** A short cervix <25 mm at 16 weeks was associated with a higher prevalence of *L. iners* (9/15, 60%) than longer cervical length (45/146, 31%, *P =* 0.04, two-tailed Fisher’s exact). **c** *L. iners* dominance was associated with early preterm birth <34^+0^ weeks (12/18, 67%), but not late preterm birth, 34^+0^ to 36^+6^ weeks (5/16, 31%) or term birth (37/127, 29%, *P =* 0.003). A greater proportion of term births had *L. crispatus* dominance at 16 weeks (63/127, 46%) than both late preterm (5/16, 31%) and early preterm births <34^+0^ weeks (2/18, 11%; *P =* 0.009; Fisher’s exact). **d** A Kaplan-Meier survival curve demonstrated that *L. iners* (*n* = 54) dominance at 16 weeks is associated with earlier gestation at delivery than a microbiome dominated by *L. crispatus* (*n* = 65, *P =* 0.02; Gehan-Breslow-Wilcoxon test)

Hierarchical clustering of species data permitted classification of samples into community state types (CSTs): I (*L. crispatus*), II (*L. gasseri*), III (*L. iners*), IV (diverse species), and V (*L. jensenii*) (Fig. 1a). The most prevalent CST observed in the patient cohort was CST I (*L. crispatus*, 40%), followed by CST III, (*L. iners*, 34%), CST II (*L. gasseri*, 10%); CST V (*L. jensenii*, 9%), and CST IV (diverse, 6%; Table 2). *L. crispatus* (CST I) was most abundant among Caucasian women (*P* = 0.008), while Black women had greater numbers of CST III (*L. iners*; *P* = 0.049 and CST IV (*P* = 0.033) (Fisher’s exact, Table 2). Short CL <25 mm was significantly associated with *L. iners* (CST III) dominance (9/15, 60%) compared to those women with a CL >25 mm (45/101, 31%; *P* = 0.04; Fisher’s exact, Fig. 1b).

**Table 2:** Distribution of community state types according to ethnicity and gestation at birth

			CST, Species									
	Total population		CST I, *L. crispatus*		CST II, *L. gasseri*		CST III, *L. iners*		CST IV, diverse species		CST V, *L. jensenii*	
*n*/*N* (%)	161	(100%)	65/161	(40%)	17/161	(11%)	54/161	(34%)	11/161	(7%)	14/161	(9%)
Ethnicity												
Caucasian	104/161	(65%)	52/104	(50%)*	13/104	(13%)	26/104	(25%)	5/104	(5%)	8/104	(8%)
Asian	27/161	(17%)	7/27	(26%)	3/27	(11%)	13/27	(48%)*	1/27	(4%)	3/27	(11%)
Black	30/161	(19%)	6/30	(20%)	1/30	(3%)	15/30	(50%)*	5/30	(17%)*	3/30	(10%)
Gestation at birth												
<34 weeks	18/161	(11%)	2/18	(11%)**	1/18	(6%)	12/18	(67%)**	1/18	(6%)	2/18	(11%)
34–37 weeks	16/161	(10%)	5/16	(31%)	2/16	(13%)	5/16	(31%)	2/16	(13%)	2/16	(13%)
>37 weeks	127/161	(79%)	58/127	(46%)	14/127	(11%)	37/127	(29%)	8/127	(6%)	10/127	(8%)

*CST* community state type based on ward HCA of species data

*P < 0.05, **P < 0.01; for comparison of birth <34 vs. >34 weeks, two-tailed Fisher’s exact

### The association of the vaginal microbiome at 16 weeks of gestation and risk of preterm birth

Major differences were detected in vaginal microbial communities at 16 weeks in women subsequently delivering early preterm (<34^+0^ weeks) compared those delivering late preterm (34^+0^ to 36^+6^ weeks) or at term (>37^+0^ weeks) (Fig. 1b, Table 2). Specifically, an *L. iners*-dominated microbiome was significantly over-represented in women delivering <34^+0^ weeks (67%) compared to late preterm (31%) and term (29%; *P =* 0.003, Fisher’s exact). In contrast, *L. crispatus* dominance associated with subsequent term birth (46 vs. 11% early preterm birth; *P =* 0.009, Fisher’s exact, Fig. 1c, Table 2), and comparatively longer duration of pregnancy than *L. iners* Fig. 1d. A logistic regression mixed-effects model demonstrated that the association between gestation at birth and CST at 16 weeks persisted after accounting for ethnicity, maternal age, BMI, and gestation at sampling (*P* = 0.04; ANOVA; Additional file 8). When individual species were assessed by mixed-effects modeling following correction for potential confounders, both *L. crispatus* and *L. iners* were significantly correlated with birth outcome with *L. crispatus* positively associated with delivery >34 weeks (*P* = 0.009, *q* = 0.048) and *L. iners* positively associated with delivery <34 weeks (*P* = 0.001, *q* = 0.006; Additional file 9).

The analysis of outcomes stratified by ethnicity did not identify a significant difference between groups although this may be due to relatively small sample sizes. The majority of women delivering >34 weeks with *L. crispatus* dominance (CST I) were Caucasian (Caucasian 50/95, 53%, Asian 7/23, 30%, and Black women 6/25, 24%), but this was not significant. In those women delivering <34 with *L. iners* dominance (CST III), similar proportions were represented across ethnic groups (Caucasian 6/9, 67%, Asian 3/4, 75%, and Black women 3/5, 60%; Additional file 10).

Calculation of predictive accuracies for preterm birth using CST assignments at 16 weeks provided sensitivity and specificity values comparable to screening using cervical length [32]: *L. iners* dominance predicted preterm birth <34^+0^ weeks with 67% sensitivity and 71% specificity (Table 3). While its absence provided a 94% negative predictive value (NPV), the PPV of *L. iners* dominance for preterm birth <34 weeks was 22%. High relative *L. crispatus* abundance was strongly predictive of birth >34 weeks gestation (89% specificity and 97% PPV; Table 3).

**Table 3:** Predictive accuracies of microbial species dominance at 16 weeks for gestation <34 weeks

CST	Species	Preterm birth <34 weeks				Birth >34 weeks			
		Sens/DR (%)	Spec (%)	PPV (%)	NPV (%)	Sens/DR (%)	Spec (%)	PPV (%)	NPV (%)
I	*L. crispatus*	11	56	3	83	44	89	97	17
II	*L. gasseri*	6	89	6	88	11	94	94	12
III	*L. iners*	67	71	22	94	29	33	78	6
IV	Diverse	6	93	9	89	7	94	91	11
V	*L. jensenii*	11	92	14	89	8	89	86	11

*CST* community state type based on ward HCA of species data, *Sens*/*DR* sensitivity or detection rate, *Spec* specificity, *PPV*/*NPV* positive/negative predictive values

### Effect of progesterone intervention on vaginal microbial communities in high-risk pregnancy

Given the significant association between vaginal microbial composition at 16 weeks and cervical length and/or subsequent preterm birth <34^+0^ weeks, we next conducted a longitudinal study of the vaginal microbiome in women receiving vaginal progesterone supplementation for a short cervix (<25 mm). A total of 67 pregnant women were eligible and consented to recruitment, of which 25 were found to have a short CL <25 mm and received progesterone until 34 weeks of gestation. The remaining 42 women did not experience cervical shortening or receive any subsequent preventative intervention, and hence were used as controls. Demographics of the two groups were comparable although, as per study design, the mean CL at commencement of progesterone was significantly lower in the “short CL” than the “normal” control group (22 vs. 32 mm, *P <* 0.05) at comparable screening gestations (15^+5^ vs. 15^+0^, respectively; Table 4). Respective rates of preterm birth (<37 weeks) were higher in the progesterone (32%, 8/25) versus control groups (5%, 2/42; *P =* 0.004). A total of 234 high vaginal samples were collected from longitudinal follow-up (22, 28, and 34 weeks) at matched gestational ages among groups (Additional file 11).

**Table 4:** Participant demographics for control and progesterone groups

	High risk controls	Progesterone	Total
*n*/*N* (%)	42/67 (63%)	25/67(37%)	67/67 (100%)
Age, years			
Mean ± SD (range)	32 ± 5.5 (21–40)	32 ± 3.9 (22–38)	32 ± 5.0 (21–40)
BMI			
Mean ± SD (range)	24.7 ± 5.3 (19–48)	25.2 ± 4.7 (18.4–35)	24.9 ± 5.0 (18.4–48)
Ethnicity, *n*/*N* (%)			
Caucasian	32/42 (76%)	18/25 (72%)	50/67 (75%)
Asian	4/42 (10%)	3/25 (12%)	7/67 (10%)
Black	6/42 (14%)	4/25 (16%)	10/67 (15%)
Smoker *n*/*N* (%)	2/42 (5%)	0/25 (0%)	2/67 (3%)
Screening for progesterone			
GA (weeks), median, range	15^+0^ (12^+1^–18^+2^)	15^+6^ (12^+0^–18^+6^)	15^+3^ (12^+0^–18^+6^)
CL (mm), median, range	32 (26–43)	22 (13–25)	(13–43)
Gestation at delivery, *n*/*N* (%)			
Early PTB, <34^+0^ weeks	1/42 (2%)	4/25 (16%)	5/67 (7%)
Late PTB, 34^+0^ to <37^+0^ weeks	1/42 (2%)	4/25 (16%)	5/67 (7%)
Term, ≥37^+0^ weeks	40/42 (95%)	17/25 (68%)	57/67 (85%)

*PTB* preterm birth, *GA* gestational age, *CL* cervical length (mm)

**Fig. 2:**
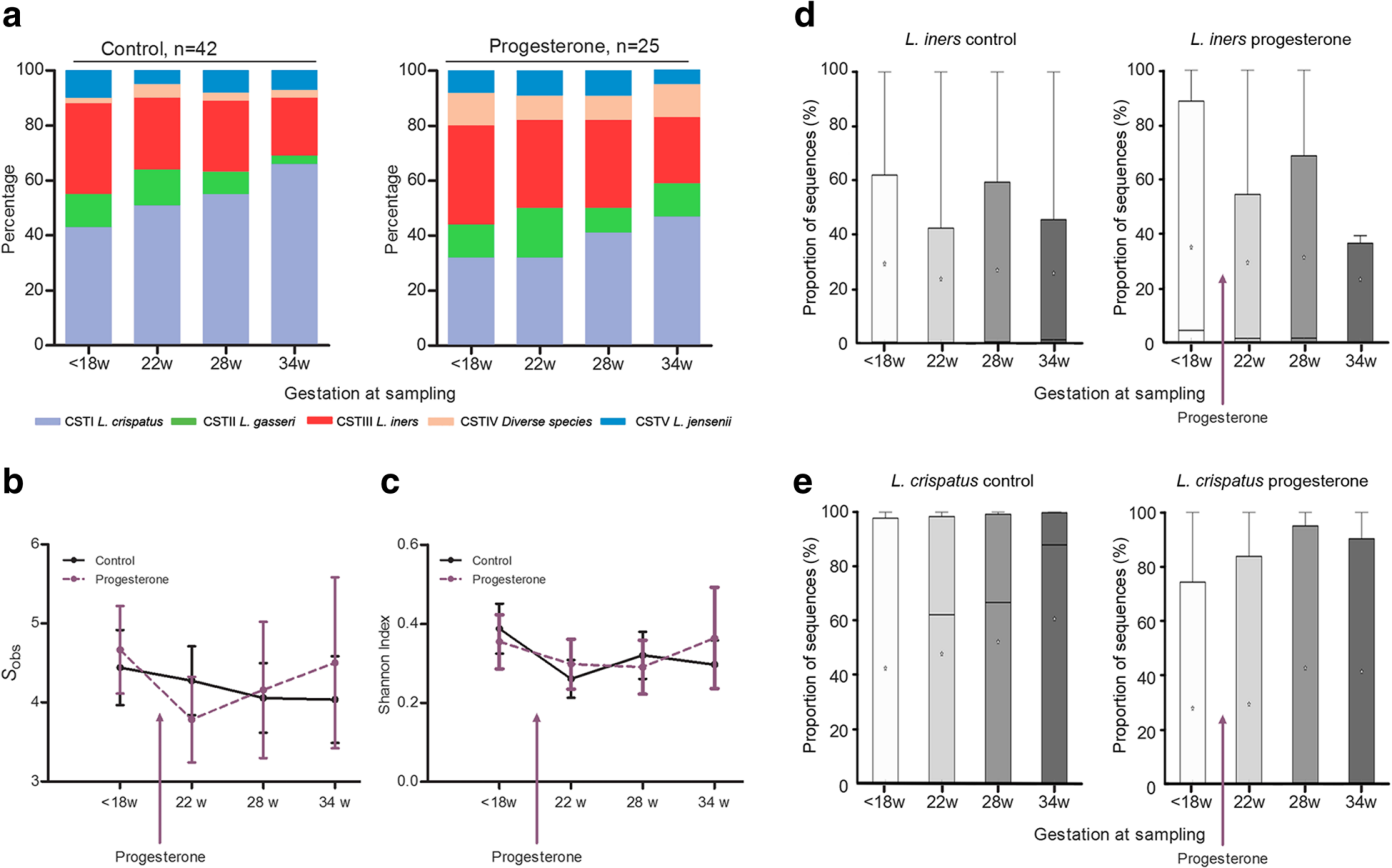
Vaginal progesterone treatment does not alter structure of the vaginal microbiome. **a** Compared to controls (*n* = 42), progesterone supplementation (*n* = 25) had no significant impact upon microbial community profiles with advancing gestation. Similarly, no effect of progesterone treatment upon **b** the number of species observed or **c** the corresponding Shannon index of alpha diversity was observed (2-way ANOVA). Fewer women requiring progesterone had a *L. crispatus* dominated microbiome compared to controls (8/25, 32 vs. 18/42, 43%, *P =* 0.4); however, progesterone treatment was associated with increased relative *L. crispatus* abundance with advancing gestation. Advancing gestational age from 18 to 34 weeks was not associated with a significant shift in mean relative abundance of *L. iners* (**d**) or *L. crispatus* (**e**) in either the controls or progesterone groups (Kruskal-Wallis, Dunn’s multiple comparison)

The distribution of CSTs in the progesterone and control groups at each sampling time point is provided in Additional file 12. Prior to progesterone intervention, no significant difference in the distribution of CSTs between to the two patient cohorts was observed (Fig. 2; Additional file 12). Vaginal progesterone supplementation had no effect upon vaginal bacterial community state structure throughout pregnancy (Fig. 2a) nor were species richness or alpha diversity measurements altered (Fig. 2b, c). Progesterone supplementation did not significantly impact on mean relative abundance of *L. iners* or *L. crispatus* with advancing gestation when compared to controls (Fig. 2d, c; Additional file 13).

**Fig. 3:**
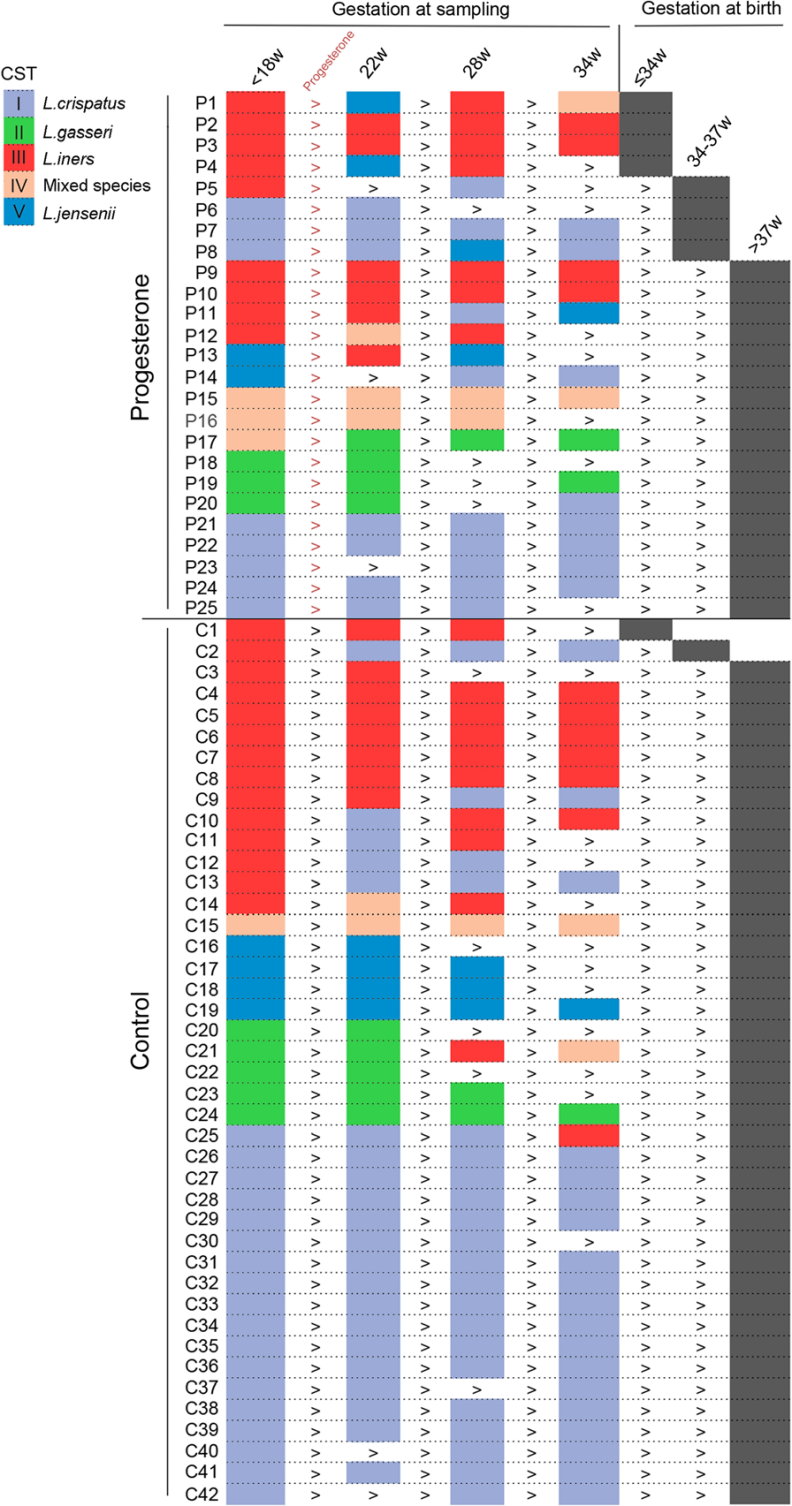
Longitudinal profiling of community state types for progesterone (*n* = 25) and control groups (*n* = 42). Progesterone supplementation was commenced after the first sampling time point (<18 weeks). Each longitudinal sample was assigned to a CST (Fig. 1a) as indicated by the *color-coded rectangle* and categorized as a function of delivery gestation

The dynamics of individual vaginal CSTs during pregnancy were then longitudinally assessed in both progesterone and control cohorts (Fig. 3). Regardless of intervention, a *L. crispatus* (CST I)*-*dominated microbiome was associated with high stability throughout pregnancy with 92% (24/26) of women maintaining *L. crispatus* dominance across all sampling time points. In contrast, significantly lower stability was observed in the 23 women exhibiting a *L. iners-*dominated microbiome at the first sampling with 17 (74%) of these women experiencing a shift to an alternative CST at some stage during their pregnancy (*P <* 0.0001). Similar levels of CST-shifting were observed in those women receiving vaginal supplementation (9/25; 36%) and control patients (10/43; 23%) (*P =* 0.3).

When gestational age at sampling, maternal age, BMI, ethnicity, and cohort were incorporated into a linear mixed-effects model, progesterone treatment did not have a significant impact upon CSTs apart from CST II; however, this difference did not withstand multiple testing correction (Additional file 14). When further assessed by relative abundance of individual species, there were no significant differences in proportions of *L. gasseri*, or any other species in the control compared to progesterone cohorts (Additional file 15).

**Fig. 4:**
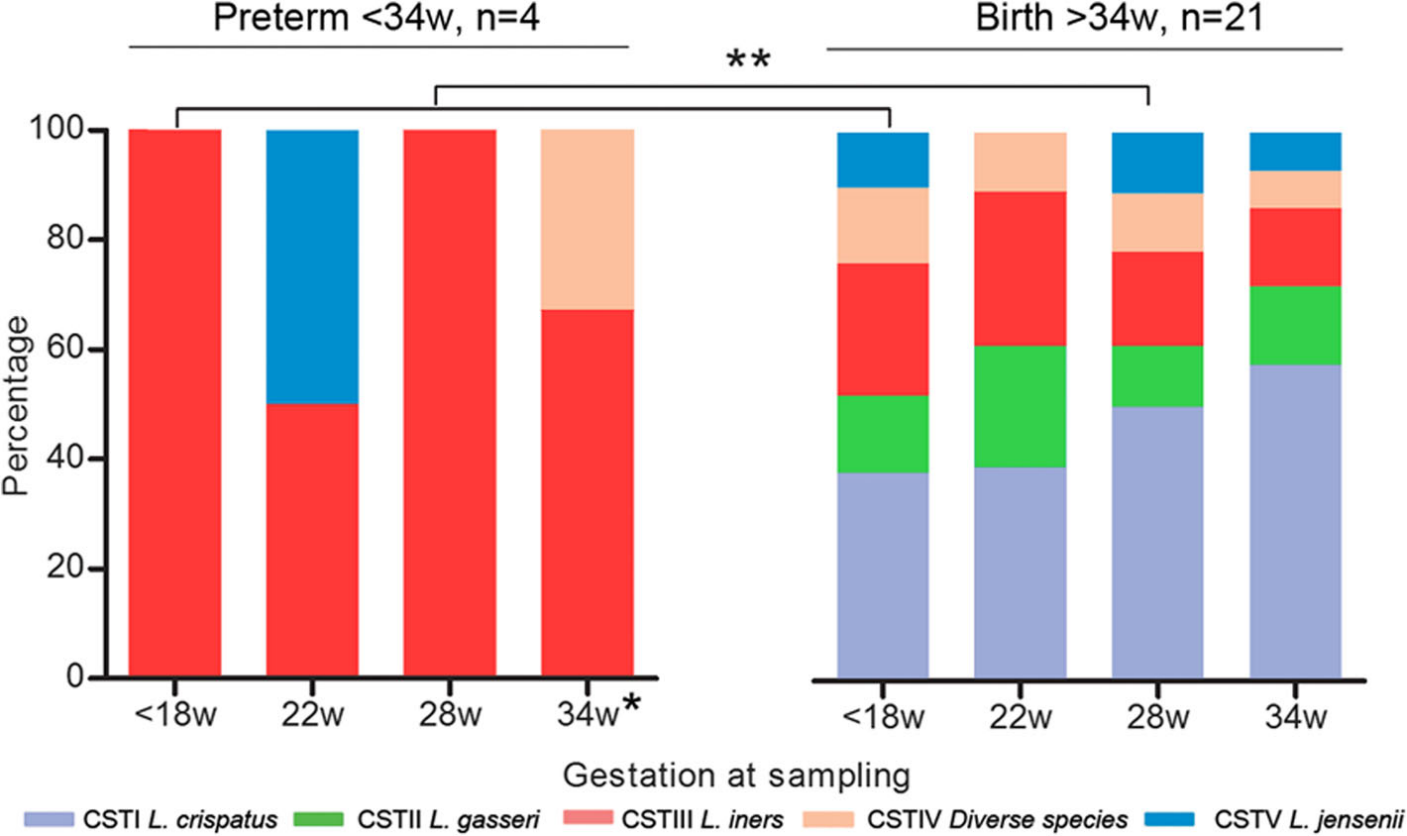
Preterm birth, despite vaginal progesterone, is associated with *L. iners* dominance throughout pregnancy. Longitudinal sampling of 25 women receiving progesterone for a short cervix showed *L. iners* dominance was associated with all women who subsequently delivered preterm <34^+0^ weeks (*n* = 4; (**P < 0.05; Fisher’s exact). *Single asterisk* indicates the delivery samples collected within 2 weeks of delivery between 28 and 34 weeks

In women receiving progesterone, marked differences in the longitudinal CST distributions were observed in women delivering <34 weeks compared to those delivering >34 weeks (Fig. 4). At 18 weeks of gestation, *L. iners* dominance was observed in 100% (4/4) of women who subsequently delivered <34 weeks of gestation compared to 24% (5/21) in women delivering >34 weeks. At delivery, *L. iners* dominance was observed in 50% (2/4) at 22 weeks and 100% (4/4) at 28 weeks (Figs. 3 and 4).

## Discussion

This study represents the largest next generation sequencing-based analysis of vaginal microbiota in pregnancies at risk of preterm birth to date. We demonstrate a significant association between *L. iners* dominance of the vaginal microbiome at 16 weeks of gestation with subsequent preterm birth and conversely show that *L. crispatus* dominance correlates with reduced risk or preterm birth. Moreover, we show that the insertion of a progesterone pessary for prevention of preterm birth has no adverse impact on vaginal microbial communities.

A healthy vaginal microbiome in non-gravid and gravid subjects is often described as being synonymous with low bacterial diversity and *Lactobacillus* species dominance [63–66]. Examination of vaginal microbiota at the time of delivery using culture and/or microscopy-based techniques has shown that *Lactobacillus* species dominance is negatively associated with delivery before 37 weeks of gestation (odds ratio 0.2) whereas bacterial dysbiosis is positively associated with preterm delivery (odds ratio 2.3) [66]. Using similar methodology, Donders and colleagues recently reported that a lactobacilli-dominated vaginal microbiome in the first trimester was associated with a 75% lower risk of delivery before 35 weeks of gestation (0.26; 95% confidence interval (CI) 0.12–0.56] compared to women harboring a vaginal microbiome void of *Lactobacillus* species (OR 2.4; 95% CI 1.2–4.8) [67]. Using culture-independent characterization of vaginal bacterial communities in a high-risk pregnant population, we show that the perceived benefit of lactobacilli dominance in pregnancy is species specific; *L. crispatus* is advantageous and associated with term delivery whereas *L. iners* is associated with increased risk of preterm delivery. Furthermore, *L. iners* is associated more specifically with a risk of early (<34 weeks) rather than late (34–37 weeks) preterm birth. High relative abundance of *L. crispatus* is highly specific for term birth, with a false positive rate (1 specificity) of just 3% in our population of women at high risk because of a previous preterm birth. In this population, second trimester dominance of *L. iners* carries a 67% detection rate (i.e., sensitivity) for preterm birth before 34 weeks; a screening sensitivity comparable to cervical length, the current and primary screening tool used for preterm birth surveillance [32, 68]. Consistent with our findings, Petricevic and colleagues recently reported an over-representation of *L. iners* dominance in vaginal swab samples collected from 13 preterm births derived from a low-risk cohort of 111 pregnancies, and none of whom delivered before 33 weeks of gestation [25]. However, this study was limited by the use of denaturing gradient gel electrophoresis (DGGE) for the characterization of only major *Lactobacillus* species and could not identify other pathobionts in the samples.

While our study reveals a clear relationship between relative abundance of vaginal *Lactobacillus* species and risk of subsequent preterm birth, our data indicate that *Lactobacillus* spp. depletion or vaginal dysbiosis in the second trimester does not appear to contribute to preterm birth risk. The role of early gestational vaginal dysbiosis in the pathology of preterm birth is controversial. In agreement with our findings, a recent longitudinal analysis of the vaginal microbiome by Romero and colleagues in 18 women experiencing preterm birth (<34 weeks gestation) reported no association between preterm birth and vaginal microbial dysbiosis when compared to controls experiencing term delivery (*n* = 72) [24]. However, in their study, 95% (17/18) of preterm birth samples and 86% of control samples were collected from African American women who exhibit a higher pregnant and non-pregnant background prevalence of vaginal dysbiosis (CST IV) [13, 24]. In contrast to these findings, Digiulio and co-workers [19] reported, in a small yet densely sampled cohort of women experiencing preterm birth (*n* = 15), which vaginal bacterial diversity does correlate with risk of preterm delivery [19]. The clinical relevance of these findings however are difficult to establish considering the small sample size and the heterogeneous nature of the cohort; only five women delivered preterm spontaneously and almost half delivered within 1 week of term dates (>36 weeks^+3/7 days^).

Our presented data provide some suggestion that vaginal microbiota in Black women may not play as an important contributory role to preterm birth pathogenesis as Caucasians and Asians. We did not however have sufficient power to demonstrate the significance of this, but this may be worth examining in future studies.

In our study, we also observed a high rate of CST-shifting in women with an *L. iners-*dominated microbiome in the second trimester compared to women with an *L. crispatus*-dominated microbiome. *L. iners* has been reported as an intermediary between lactobacilli dominance and CST IV-associated states and is the predominant microbiome in peri-menopausal women as they transition through to postmenopausal dominance of anaerobic bacteria [16]. Interactions between *L. iners* and the maternal host likely provides a vaginal mucosal environment permissible to colonization by BV-associated pathogens, a setting in which it tolerates co-existence well [69, 70]. Unlike other *Lactobacillus* species, *L. iners* also induces secretion of pro-inflammatory cytokines when human vaginal epithelial cells are observed in vitro, whereas *L. crispatus* does not [71, 72].

Other evidence for a role of bacterial dysbiosis in the pathology of preterm birth includes the long recognized association between bacterial vaginosis (BV) and increased risk of preterm birth; however, evidence suggests that screening and treating BV in pregnancy reduces preterm delivery in certain cohorts [73], but not in others [74]. We propose an alternate concept, which is that it is the presence of *L. iners* that promotes risk of early preterm birth, but because an *L. iners-*dominated vaginal microbiome has less stability, there is a tendency for transition to BV-associated CST-IV [69, 75]. Indeed, *L. iners* is the prominent vaginal species following antibiotic treatment for BV [76]. Older studies aimed at detecting BV, which could not differentiate *Lactobacillus* species and concluded that it was BV rather than species of *Lactobacillus* that conferred the risk.

Recent investigations into the protective role of *Lactobacillus* species in the context of reproductive health have revealed major species-specific differences in the capacity to prevent pathobiont colonization and viral infections [12, 72, 77, 78] that are driven largely by maternal host-bacterial metabolite interactions at the vaginal mucosal interface. For example, although lactic acid-producing bacteria including *Lactobacillus* spp. produce both the d- and l-lactic acid isomers [79], the chirality of the isomer has major functional implications. In women exhibiting a vaginal microbiome dominated by *L. iners*, an increased ratio of l to d-lactic acid has previously been shown to promote expression of vaginal extracellular matrix metalloproteinase inducer (EMMPRIN) and the activation of matrix metalloproteinase-8 (MMP8), which may subsequently modulate cervical integrity [80]. Conversely, no such relationship has been observed in women with vaginal microbial communities dominated by *L. crispatus*, which instead preferentially excretes high levels of d-lactic acid and greater overall levels of lactic acid than *L. iners* [80]. Apart from modulating local tissue inflammation, recent studies have also implicated lactate isomers in vaginal mucosal trapping mechanisms. High concentrations of d-lactic acid are associated with *L. crispatus* dominance and enhanced trapping of HIV-1 virions in cervico-vaginal mucosa whereas low concentrations of d-lactic acid associated with *L. iners* dominance permits comparatively rapid diffusion of virions through cervico-vaginal mucosa [77]. Collectively, these data suggest mechanisms by which *L. iners* dominance of vaginal microbial communities during pregnancy may lead to the modulation of local tissue inflammation and remodeling pathways and to disruption of chemical and mechanical mucosal barriers protective against ascending infection and increase the risk of preterm birth. Such mechanisms may account for the observed association between *L. iners* dominance and a short cervical length (<25 mm) seen in our study at 16 weeks of gestation, which itself is highly specific for preterm birth [81].

Considering the potential pro-inflammatory roles played by *L. iners* in the vagina during pregnancy, we postulated that any associated poor pregnancy outcomes might be attenuated by the anti-inflammatory actions of progesterone [45, 82]. However, in this study, no effect of vaginal progesterone therapy upon the frequency of vaginal community state structure was observed across pregnancy indicating that the mode of action of progesterone in the prevention of preterm birth is not through modulation of the vaginal microbiome. The data also show, however, that there is no detrimental effect upon the vaginal microbiome of either progesterone itself or of the daily vaginal insertion of a pessary.

A particular strength of our study is that we characterized the vaginal microbiome in a comparatively large patient cohort at high risk of preterm birth. This strength was demonstrated by a high spontaneous preterm birth rate (*n* = 34/161). The mean gestation at birth of 32^+6^ weeks within our preterm birth cohort, and a distribution of gestational ages ranging from 24 to 36 weeks, enabled the characterization of microbial profiles associated with both early (<34 weeks) and late (34 > 37 weeks) preterm birth, providing a broader observational base for microbial-host interactions in pregnancy. The primary limitation was the small number of women receiving progesterone (*n* = 25) and the lack of an equivalent control group with a short CL <25 mm not receiving any intervention or receiving a placebo. A short CL significantly increases subsequent preterm birth risk [33]; therefore, once detected, clinicians are ethically obliged to provide a preventative intervention such as progesterone. Consequently, a “placebo” intervention for a short cervix could not be included for study in this clinical study. As such, the control women are not true controls as their cervical lengths were all greater than 25 mm at entry. A further potential confounding factor was the impact of ethnicity of vaginal microbiota and gestation at birth, although we demonstrated this not to be significant in our cohort.

## Conclusions

Our data indicate that specific *Lactobacillus* species have differing associations with outcome in pregnancies at high risk of preterm birth. Detection of vaginal microbial composition in the early second trimester may be used to stratify preterm birth risk; *L. crispatus* dominance is highly predictive of term birth, while high *L. iners* relative abundance is associated with increased risk of preterm birth and warrants heighted surveillance. Increased diversity of vaginal microbiota at 16 weeks of gestation is not associated with increased risk of preterm birth. The use of progesterone therapy for preterm birth prevention does not appear to adversely affect the relative abundance of vaginal *Lactobacillus* species or species diversity, indicating that progesterone’s mode of action during pregnancy is likely not via modulation of vaginal microbial communities. Patients and clinicians who may be concerned about the “infection risk” associated with the use of vaginal pessaries during high-risk pregnancy can be reassured.

## Additional files

Additional file 1: Work flow of methodology for cross-sectional and longitudinal studies.
Additional file 2: Detailed methodology for statistical analyses.
Additional file 3: Species read count data for cross-sectional cohort.
Additional file 4: Patient metadata for cross-sectional cohort.
Additional file 5: Species read count data for longitudinal cohort (progesterone treatment).
Additional file 6: Patient metadata for longitudinal cohort (progesterone treatment).
Additional file 7: Preterm birth does not associate with vaginal dysbiosis at 16 weeks of gestation. (A) Heatmap of ward hierarchical clustering of microbial genera from 161 women sampled at 16 weeks of gestation, classified according to subsequent gestation at delivery. Women delivering preterm both <34^+0^ weeks (*n* = 18, red) and 34^+0^ to 36^+6^ weeks (*n* = 16, orange) had a predominantly *Lactobacillus* species-dominated vaginal microbiome, as did women experiencing term births >37^+0^ weeks (*n* = 127, gray). (B) No correlation between richness (number of bacterial species observed; *S*_obs_) (C), nor alpha diversity as measured using the Shannon index with gestation at birth was observed. (*ns* non-significant, 2-way ANOVA).
Additional file 8: Linear mixed-effects model of cross-sectional data assessing the correlation between CST, gestational age at sample, maternal age, and BMI on pregnancy outcome.
Additional file 9: Linear mixed-effects model assessing the impact of relative species abundance at 16 weeks on subsequent gestation at birth.
Additional file 10: Distribution of CSTs as a function of ethnicity and gestation at birth in cross-sectional cohort.
Additional file 11: Gestational age at vaginal sampling in high-risk control and progesterone groups.
Additional file 12: Community state type classification of samples as a function of gestation at sampling: progesterone versus control groups.
Additional file 13: Comparison of mean *L. iners* and *L. crispatus* relative abundance in control (*n* = 42) versus progesterone groups (*n* = 25) and as a function of birth before and after 34 weeks. (A) Prior to progesterone intervention at <18-week sampling, women with a short CL <25 mm had greater relative abundance of *L. iners* compared to controls, and lower *L. crispatus* (B) although this did not reach significance. *L. iners* abundance declined in both control and progesterone groups towards 34 weeks of sampling while mean *L. crispatus* abundance increased (ANOVA, K-W, Dunn’s multiple comparison). Inclusive of control and progesterone groups, preterm birth <34 weeks was associated with higher mean *L. iners* abundance at longitudinal sampling (C; *P <* 0.05), and lower mean *L. crispatus* abundance (D; *P <* 0.001) than deliveries >34 weeks, at matched gestational age at sampling throughout follow-up (Welch’s *t* test).
Additional file 14: Mixed-effects model assessing the impact of progesterone treatment on CST profile at longitudinal sampling, incorporating potential contributing confounders (gestational age at sample, maternal age, BMI, and ethnicity).
Additional file 15: Mixed-effects model comparing relative species abundance in control and progesterone groups.

## Abbreviations

bp: Base pairs
BV: Bacterial vaginosis
CI: Confidence interval
CL: Cervical length
CST: Community state type
DDGE: Denaturing gradient gel electrophoresis
EMMPRIN: Extracellular matrix metalloproteinase inducer
HCA: Hierarchical cluster analysis
HIV: Human immunodeficiency virus
MMP: Metalloproteinase
NHS: National Health Service
NPV: Negative predictive value
OD: Omne in die (once per day)
OR: Odds ratio
PPV: Positive predictive value
RDP: Ribosomal database project
REC: Research ethics committee
rRNA: Ribosomal ribonucleic acid
SD: Standard deviation
STAMP: Statistical Analysis of Metagenomic Profiles
TVS: Transvaginal scan
