# Supplementary material for: The interaction between vaginal microbiota, cervical length, and vaginal progesterone treatment for preterm birth risk
Source: Microbiome. 2017 Jan 19;5:6. doi: 10.1186/s40168-016-0223-9 (PMC5244550; doi:10.1186/s40168-016-0223-9)
Supplement: Additional file 1: — Work flow of methodology for cross-sectional and longitudinal studies. [file 40168_2016_223_MOESM1_ESM.docx]

**Work Flow**

Cross sectional study

Longitudinal study
