# Supplementary material for: The interaction between vaginal microbiota, cervical length, and vaginal progesterone treatment for preterm birth risk
Source: Microbiome. 2017 Jan 19;5:6. doi: 10.1186/s40168-016-0223-9 (PMC5244550; doi:10.1186/s40168-016-0223-9)
Supplement: Additional file 2: — Detailed methodology for statistical analyses. [file 40168_2016_223_MOESM2_ESM.docx]

**Additional File 2. Detailed methodology for statistical analyses.**

This document contains details for statistical analyses conducted in Stamp (Statistical Analysis of Metagenomic Profiles) and also the R environment for the manuscript, “The interaction between vaginal microbiota, cervical length and vaginal progesterone treatment for preterm birth risk. Lindsay M. Kindinger, Phillip R. Bennett, Yun S Lee, Julian R. Marchesi, Ann Smith, Stefano Cacciatore, Elaine Holmes, Jeremy K. Nicholson, TG Teoh and David A. MacIntyre. 2016. Microbiome.

All data required to reproduce analysis can be found here: <https://github.com/tkcaccia/vaginal-microbiota>. These data are also provided as Supplementary files to the main manuscript (Additional files 12-15).

**Does vaginal microbiota in high-risk pregnancy differ according to ethnicity, short cervical length, need for preventative intervention and subsequent preterm birth?**

Statistical Analysis of Metagenomic Profiles (STAMP) software package [60] was downloaded from <http://kiwi.cs.dal.ca/Software/STAMP> and version STAMP v2.1.3 installed.

As per STAMP user instructions, species level taxonomy data from individual participants and corresponding cross-sectional metadata file (provided in Additional files 12 and 13), were imported.

Heatmaps were generated in STAMP and ward linkage hierarchical clustering analysis (HCA; clustering density threshold of 0.75) performed permitting classification of samples into 1 of 5 community state types (CSTs as described by Ravel et al [13].). The total number of samples assigned to CST I (*L. crispatus*), II (*L. gasseri*), III (*L. iners*), IV (mixed bacterial species) and V (*L. jensenii*), were determined and presented as Figure 1A in the manuscript (see below).


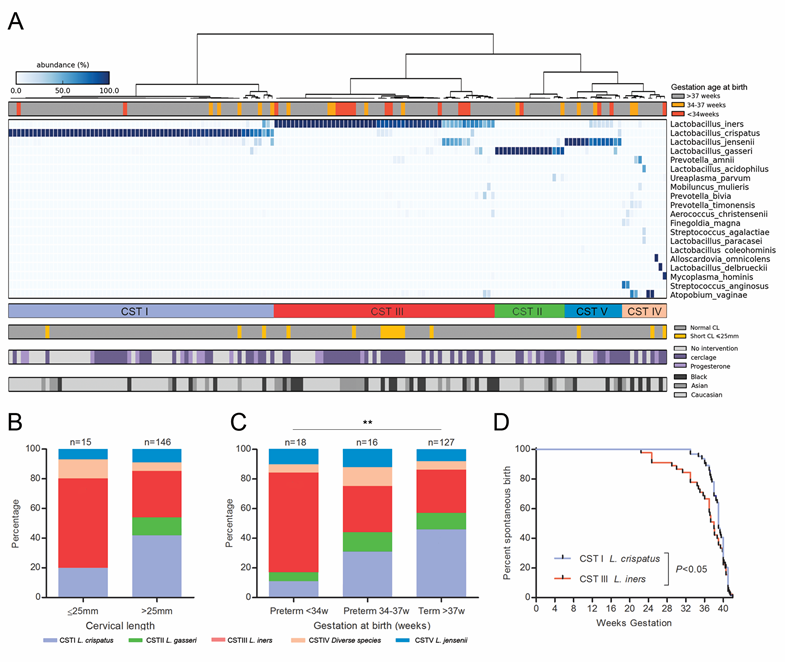


Using corresponding metadata for short cervical length, need for preventative intervention and subsequent preterm birth (Additional file 13), proportions of assigned CSTs were grouped into short and non-short CLs (Figure 1B), and categorized by eventual gestation at birth (Figure 1C) (see below). The Graphpad statistical software package was used create survival curves comparing duration of pregnancy gestation among women assigned to CST I and III at 16 weeks (Figure 1D, and below). Further details for generating these curves can be found here: https://www.graphpad.com/guides/prism/6/statistics/index.htm?stat_howto_survival.htm


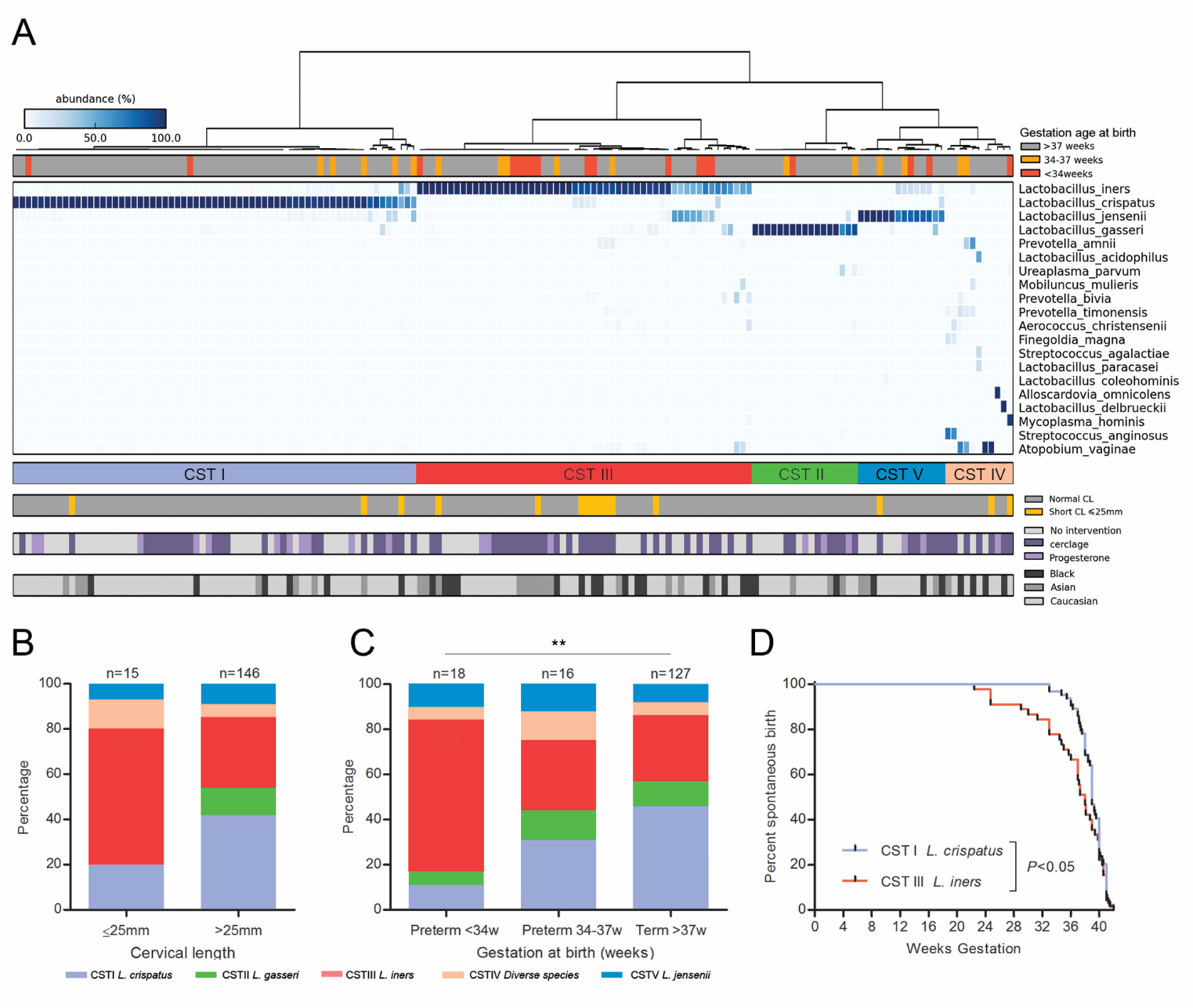


Description of logistic regression-mixed effects modeling on this data (Additional Figures 3 and 4) as performed in the R environment is detailed later in this document.

**Does insertion of a progesterone pessary impact on longitudinal vaginal microbiota?**

Women were allocated to control and progesterone groups as described in the methods. Classification of CSTs at each gestational timepoint (<18w, 22w, 28w, and 34w) in control and progesterone groups was performed in STAMP as described above using species data for each individual (Additional file 14), and corresponding metadata (Additional file 15). CST distributions (% of total), at each timepoint, were compared in the control and progesterone groups using Fisher Exact in Graph pad (Figure 2A and below).


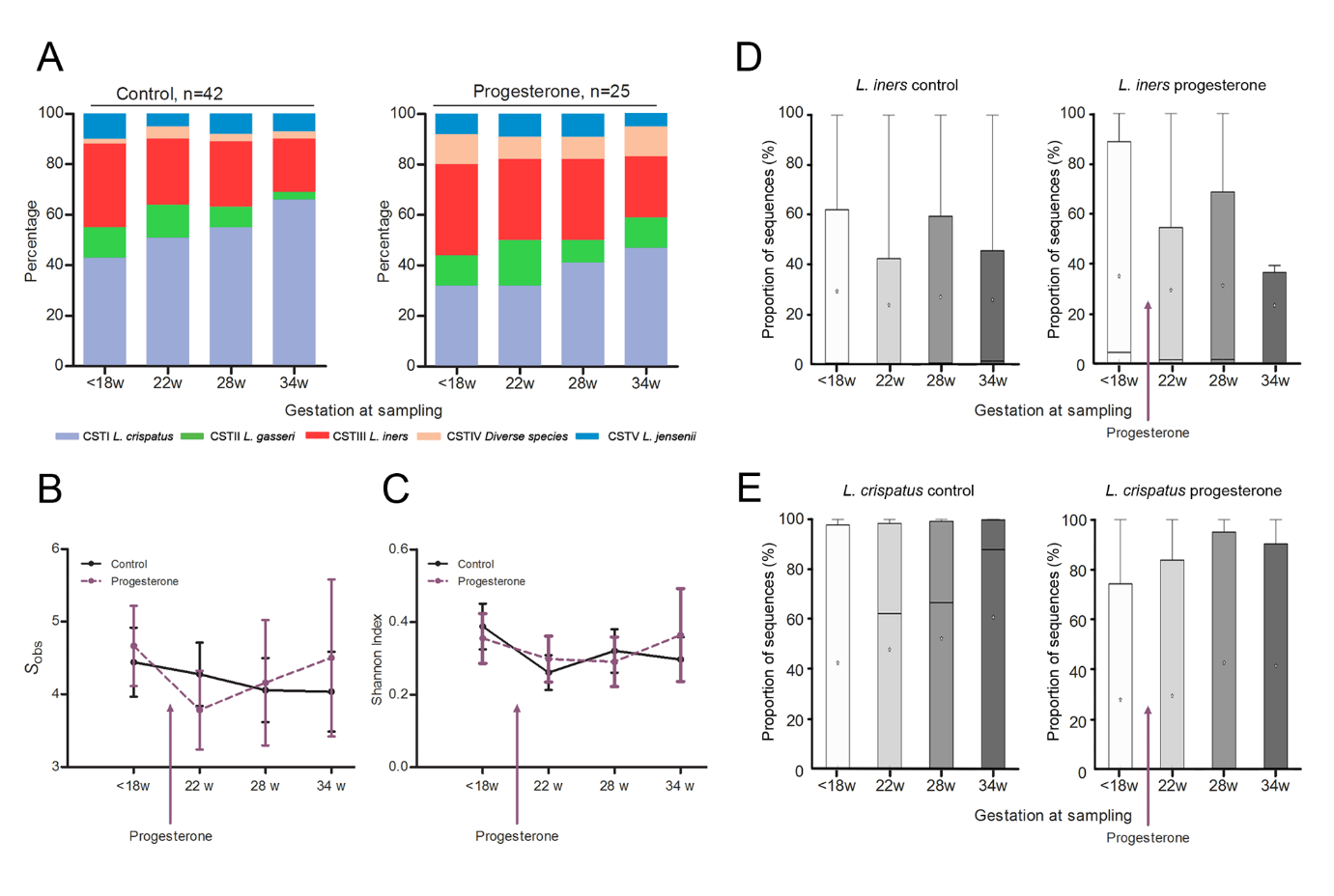


**Does insertion of a progesterone pessary impact on longitudinal proportions of *L. iners* and *L. crispatus* sequence reads?**

Sequence reads from individual samples in the longitudinal cohort (Additional File 14) were uploaded to STAMP, together with corresponding metadata for samples (Additional File 15). Box and Whisker plots were generated as output in STAMP. Sequence reads of *L. iners* for participants at timepoints <18, 22, 28 and 34weeks (as selected from corresponding metadata, Additional file 15) were plotted (mean, median and 95 percentile ranges for sequence reads). The control and progesterone groups were plotted separately in STAMP, and the associated STAMP statistician function provided *P*-values for longitudinal variations with advancing gestation (Figure 2D; ANOVA ). This was repeated for *L. crispatus* sequence reads (Figure 2D)


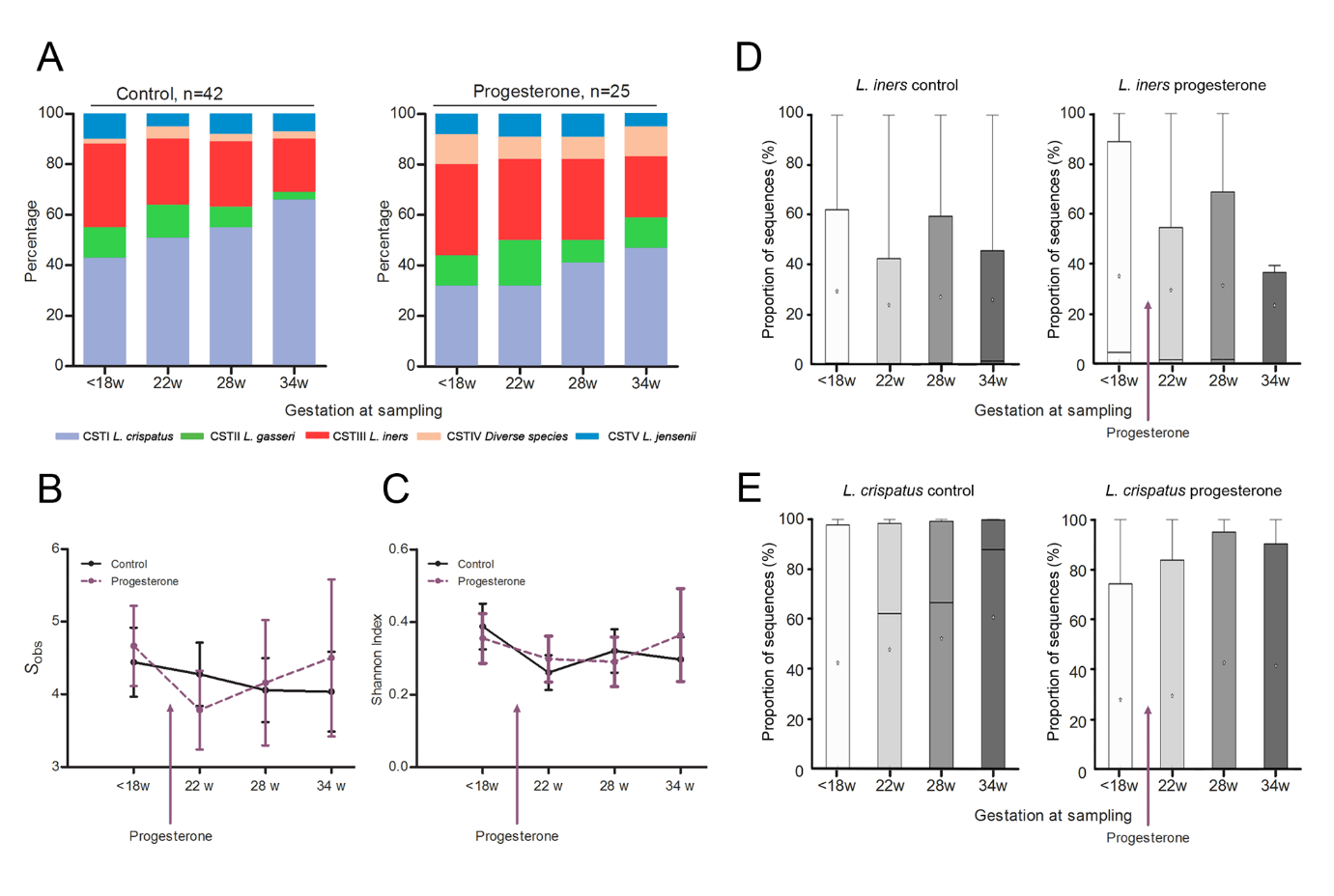


**Pictorial description of longitudinal shifts in CSTs.**

CST classifications for each patient were allocated a unique color plotted as a function of sample collection time in Microsoft Excel (Professional Plus 2013) for each individual. Subsequent categorized gestation at birth was also plotted (provided in Additional File 15)


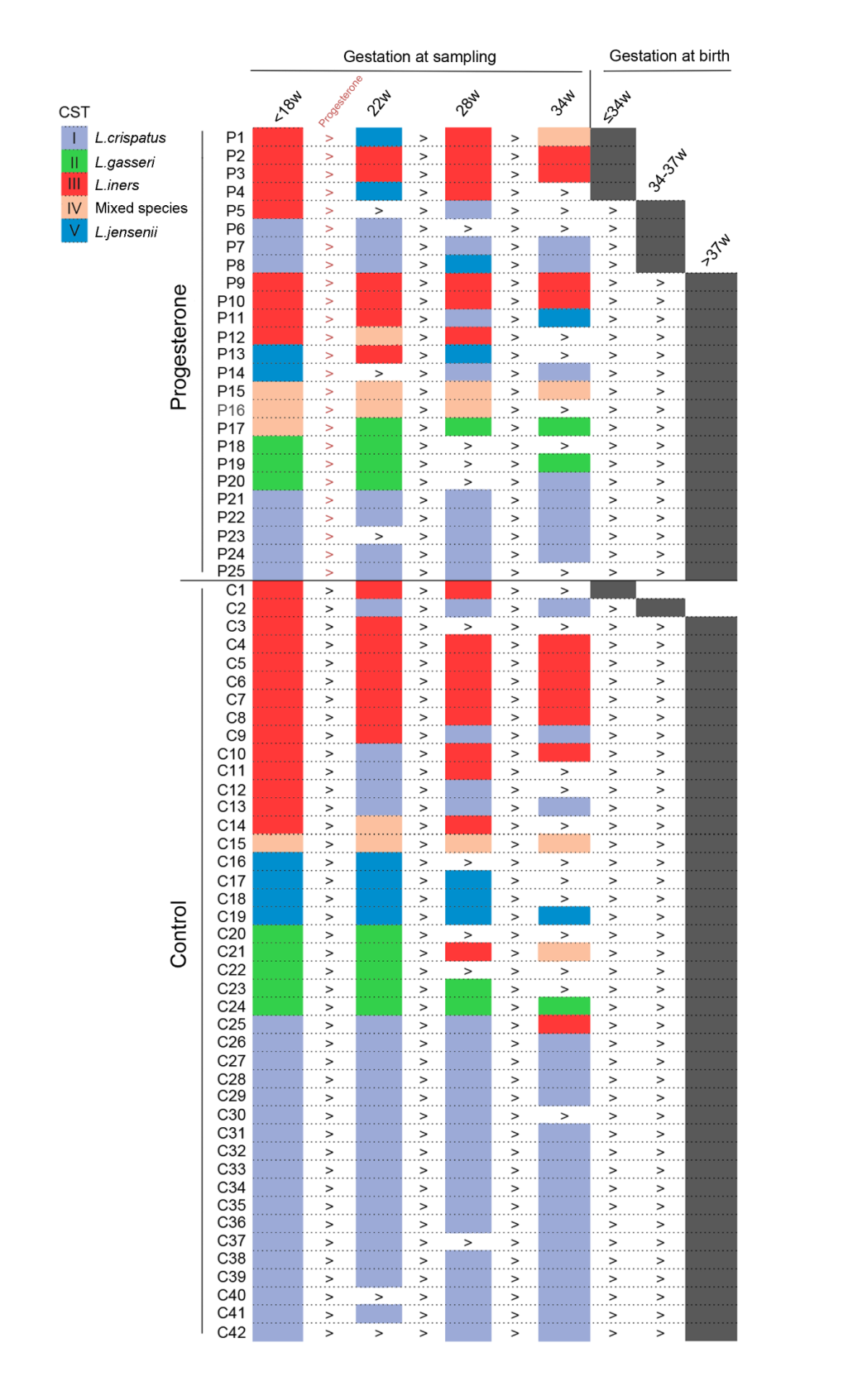


**The follow pages provide all details for mixed effects modelling performed in R.**

**Mixed-effects model of cross-sectional data assessing the correlation between CST, gestational age at sample, maternal age and BMI on pregnancy outcome (Additional File 3).**

Firstly ensure the R: The R Project for Statistical Computing programming environment is installed and appropriate libraries installed and loaded. We have used the R version 3.3.2.

> library("lmerTest")

>

> r=read.csv("Data/Prog data.csv”)

>

> r[,"Preterm.birth.outcome"]=as.numeric(r[,"Preterm.birth.outcome"])

>

> test <- glm(Preterm.birth.outcome ~

+ Gestation.at.sample+

+ Age+

+ BMI+

+ CST, data = r)

>

> anova(test, test = "Chisq")

Analysis of Deviance Table

Model: gaussian, link: identity

Response: Preterm.birth.outcome

Terms added sequentially (first to last)

Df Deviance Resid. Df Resid. Dev Pr(>Chi)

NULL 160 15.988

Gestation.at.sample 1 0.28617 159 15.701 0.08188 .

Age 1 0.24088 158 15.460 0.11043

BMI 1 0.00358 157 15.457 0.84573

CST 5 1.08803 152 14.369 0.04216 *

---

Signif. codes: 0 ‘***’ 0.001 ‘**’ 0.01 ‘*’ 0.05 ‘.’ 0.1 ‘ ’ 1

**Mixed-effects model assessing the impact of relative species abundance at 16 weeks on subsequent gestation at birth (Additional File 4).**

> library("lmerTest")

>

> d=read.csv("Data/Species data 1.csv")

>

> d[,"Preterm.birth.outcome"]=as.numeric(d[,"Preterm.birth.outcome"])

>

> da=NULL

> name=NULL

> meanbad=NULL

> meangood=NULL

> fold=NULL

> for(i in 7:56){

+

+ if(mean(as.numeric(d[,i]>0))>0.05){

+ ll=lmer(Preterm.birth.outcome~1+

+ Gestation.at.sample+

+ BMI+

+ Age+

+ d[,i]+

+ (1|Ethnicity),d)

+ w=anova(ll)

+ if(nrow(w)==4){

+ name=c(name,colnames(d)[i])

+ da=rbind(da,as.matrix(w)["d[, i]",])

+ meangood=c(meangood,mean(d[d[,"Preterm.birth.outcome"]==2,i]))

+ meanbad=c(meanbad,mean(d[d[,"Preterm.birth.outcome"]==1,i]))

+

+ }

+ }

+ }

> rownames(da)=name

> da=data.frame(before34=meanbad,after34=meangood,log2_of_the_ratio=-log2(meanbad/meangood),da,FDR=p.adjust(da[,"Pr(>F)"],method="fdr"))

> da

before34 after34 log2_of_the_ratio Sum.Sq Mean.Sq NumDF DenDF F.value Pr..F. FDR

Lactobacillus_crispatus 87.66666667 307.9020979 1.8123714 0.6687657800 0.6687657800 1 156.0000 7.054784538 0.0087267102 0.047996906

Lactobacillus_iners 439.05555556 183.3286713 -1.2599711 1.1425136659 1.1425136659 1 156.0000 12.451214499 0.0005490671 0.006039738

Lactobacillus_gasseri 40.94444444 85.3426573 1.0595993 0.0457424132 0.0457424132 1 156.0000 0.463027694 0.4972210311 0.607714594

Lactobacillus_jensenii 114.72222222 72.5314685 -0.6614659 0.0770639943 0.0770639943 1 156.0000 0.781669135 0.3779917122 0.607714594

Atopobium_vaginae 0.00000000 16.2167832 Inf 0.0621896913 0.0621896913 1 156.0000 0.630187863 0.4284922831 0.607714594

Prevotella_bivia 0.77777778 2.5104895 1.6905388 0.0166166474 0.0166166474 1 156.0000 0.167884769 0.6825606494 0.750816714

Aerococcus_christensenii 0.00000000 2.3076923 Inf 0.1309059026 0.1309059026 1 154.0214 1.333000494 0.2500603236 0.607714594

Prevotella_timonensis 0.00000000 1.6643357 Inf 0.1440972319 0.1440972319 1 156.0000 1.467993377 0.2274946246 0.607714594

Finegoldia_magna 0.05555556 1.1048951 4.3138344 0.0807814971 0.0807814971 1 156.0000 0.819574303 0.3667007140 0.607714594

Lactobacillus_vaginalis 0.00000000 0.2167832 Inf 0.0534939685 0.0534939685 1 156.0000 0.541765330 0.4628073512 0.607714594

Ureaplasma_parvum 1.16666667 1.8111888 0.6345445 0.0007783849 0.0007783849 1 156.0000 0.007856282 0.9294851450 0.929485145

**Mixed-effects model assessing the impact of progesterone treatment on CST profile at longitudinal sampling, incorporating contributing confounders (gestational age at sample, maternal age, BMI and ethnicity) (Additional File 9).**

> library("lmerTest")

>

> u=read.csv("Data/Prog longitudinal data.csv")

>

> u1=u

> u1[,"CST"]=as.numeric(u1[,"CST"]=="I")

> ll=lmer(CST~1+

+ Timepoint..weeks.+(0+Timepoint..weeks.|Patient.number)+

+ treatment+

+ BMI+

+ Age+

+ Cohort+

+ (1|Patient.number)+

+ (1|Ethnicity),u1)

> a=anova(ll)

>

> u2=u

> u2[,"CST"]=as.numeric(u2[,"CST"]=="II")

> ll=lmer(CST~0+

+ Timepoint..weeks.+(0+Timepoint..weeks.|Patient.number)+

+ treatment+

+ BMI+

+ Age+

+ Cohort+

+ (1|Patient.number)+

+ (1|Ethnicity),u2)

> b=anova(ll)

>

> u3=u

> u3[,"CST"]=as.numeric(u3[,"CST"]=="III")

> ll=lmer(CST~1+

+ Timepoint..weeks.+(0+Timepoint..weeks.|Patient.number)+

+ treatment+

+ BMI+

+ Age+

+ Cohort+

+ (1|Patient.number)+

+ (1|Ethnicity),u3)

> c=anova(ll)

>

> u4=u

> u4[,"CST"]=as.numeric(u4[,"CST"]=="IV")

> ll=lmer(CST~1+

+ Timepoint..weeks.+(0+Timepoint..weeks.|Patient.number)+

+ treatment+

+ BMI+

+ Age+

+ Cohort+

+ (1|Patient.number)+

+ (1|Ethnicity),u4)

> d=anova(ll)

>

> u5=u

> u5[,"CST"]=as.numeric(u5[,"CST"]=="V")

> ll=lmer(CST~1+

+ Timepoint..weeks.+(0+Timepoint..weeks.|Patient.number)+

+ treatment+

+ BMI+

+ Age+

+ Cohort+

+ (1|Patient.number)+

+ (1|Ethnicity),u5)

> e=anova(ll)

>

>

>

> tt=rbind(a["treatment",],b["treatment",],c["treatment",],d["treatment",],e["treatment",])

> fdr=p.adjust(tt[,6])

> tt=cbind(tt,fdr)

> rownames(tt)=c("CSTI","CSTII","CSTIII","CSTIV","CSTV")

> tt

Sum Sq Mean Sq NumDF DenDF F.value Pr(>F) fdr

CSTI 0.002133164 0.002133164 1 159.7697 0.04835184 0.82623713 1.0000000

CSTII 0.029863453 0.029863453 2 156.3853 4.23518634 0.04125418 0.2062709

CSTIII 0.071963747 0.071963747 1 161.6621 1.14884679 0.28538818 1.0000000

CSTIV 0.002144753 0.002144753 1 164.3165 0.08310623 0.77349457 1.0000000

CSTV 0.011838545 0.011838545 1 164.9066 0.36261045 0.54788709 1.0000000

**Mixed-effects model comparing relative species abundance in the control and progesterone groups corrected for gestational age at sample, maternal age, BMI and ethnicity (Additional File 10).**

> library("lmerTest")

>

> q=read.csv("Data/Species data 2.csv")

>

>

> da=NULL

> name=NULL

> meancontrol=NULL

> meantreated=NULL

> fold=NULL

> for(i in 11:50){

+ if(mean(as.numeric(q[,i]>0))>0.05){

+ ll=lmer(q[,i]~1+

+ Timepoint..weeks.+(0+Timepoint..weeks.|Patient.number)+

+ treatment+

+ BMI+

+ Age+

+ Cohort+

+ (1|Patient.number)+

+ (1|Ethnicity),q)

+ w=anova(ll)

+ if(nrow(w)==5){

+ name=c(name,colnames(q)[i])

+ da=rbind(da,as.matrix(w)["treatment",])

+ meantreated=c(meantreated,mean(q[q[,"treatment"]==1,i]))

+ meancontrol=c(meancontrol,mean(q[q[,"treatment"]==0,i]))

+ }

+ }

+

+ }

> rownames(da)=name

> da=data.frame(Control_group=meancontrol,Progesterone_group=meantreated,log2_of_the_ratio=-log2(meantreated/meancontrol),da,FDR=p.adjust(da[,"Pr(>F)"],method="fdr"))

> da

Control_group Progesterone_group log2_of_the_ratio Sum.Sq Mean.Sq NumDF DenDF F.value Pr..F. FDR

Lactobacillus_crispatus 300.0406977 380.8500000 -0.3440647 1.882580e+04 1.882580e+04 1 160.7329 0.3703472 0.5436741 0.693639

Lactobacillus_iners 203.8720930 163.8166667 0.3155822 6.834972e+03 6.834972e+03 1 162.5462 0.1557267 0.6936390 0.693639

Lactobacillus_gasseri 70.9767442 93.1666667 -0.3924675 3.979515e+04 3.979515e+04 1 164.8166 2.1933400 0.1405176 0.693639

Lactobacillus_jensenii 80.1976744 42.7500000 0.9076360 2.069985e+03 2.069985e+03 1 167.4654 0.1563711 0.6930230 0.693639

Atopobium_vaginae 35.7674419 13.9333333 1.3601065 1.169273e+04 1.169273e+04 1 171.0074 0.9946185 0.3200253 0.693639

Lactobacillus_vaginalis 0.3372093 0.5166667 -0.6155895 5.156162e-01 5.156162e-01 1 187.6014 0.2045481 0.6515970 0.693639
