# Supplementary figures and images for: The interaction between vaginal microbiota, cervical length, and vaginal progesterone treatment for preterm birth risk
Source: Microbiome. 2017 Jan 19;5:6. doi: 10.1186/s40168-016-0223-9 (PMC5244550; doi:10.1186/s40168-016-0223-9)

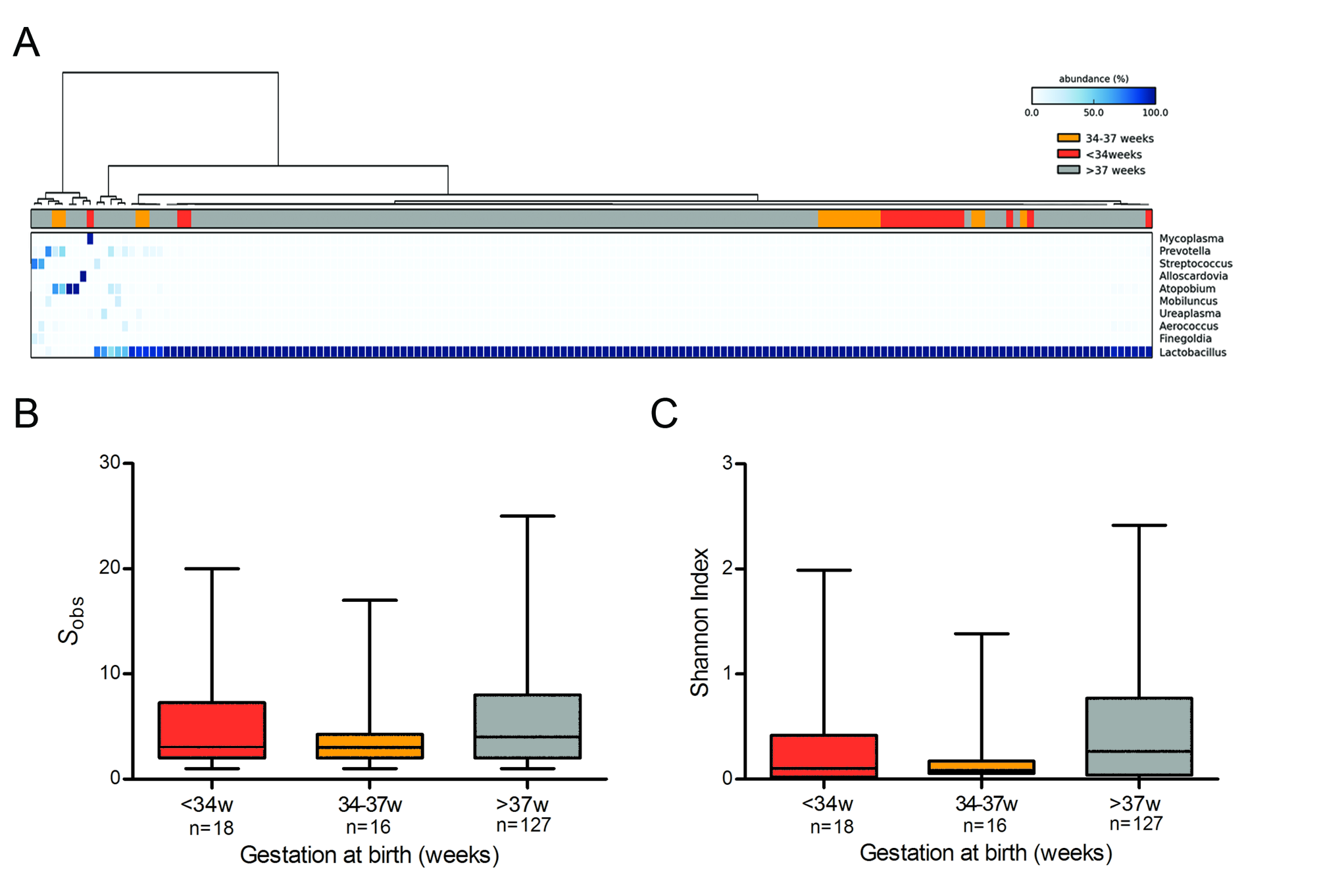

Supplement: Additional file 7: — Preterm birth does not associate with vaginal dysbiosis at 16 weeks of gestation. (A) Heatmap of ward hierarchical clustering of microbial genera from 161 women sampled at 16 weeks of gestation, classified according to subsequent gestation at delivery. Women delivering preterm both <34+0 weeks (n = 18, red) and 34+0 to 36+6 weeks (n = 16, orange) had a predominantly Lactobacillus species-dominated vaginal microbiome, as did women experiencing term births >37+0 weeks (n = 127, gray). (B) No correlation between richness (number of bacterial species observed; S obs) (C), nor alpha diversity as measured using the Shannon index with gestation at birth was observed. (ns non-significant, 2-way ANOVA). [file 40168_2016_223_MOESM7_ESM.tif]

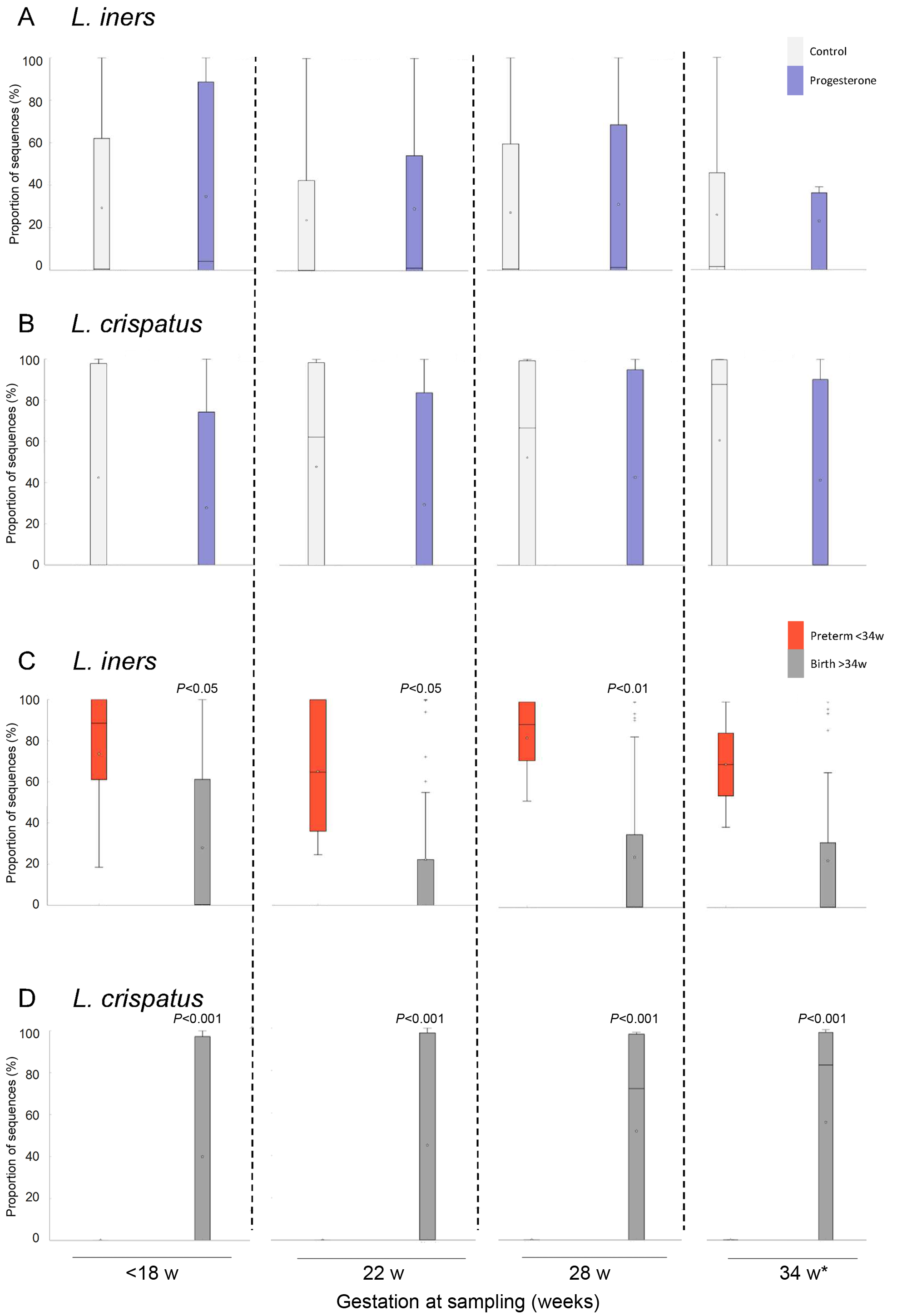

Supplement: Additional file 13: — Comparison of mean L. iners and L. crispatus relative abundance in control (n = 42) versus progesterone groups (n = 25) and as a function of birth before and after 34 weeks. (A) Prior to progesterone intervention at <18-week sampling, women with a short CL <25 mm had greater relative abundance of L. iners compared to controls, and lower L. crispatus (B) although this did not reach significance. L. iners abundance declined in both control and progesterone groups towards 34 weeks of sampling while mean L. crispatus abundance increased (ANOVA, K-W, Dunn’s multiple comparison). Inclusive of control and progesterone groups, preterm birth <34 weeks was associated with higher mean L. iners abundance at longitudinal sampling (C; P < 0.05), and lower mean L. crispatus abundance (D; P < 0.001) than deliveries >34 weeks, at matched gestational age at sampling throughout follow-up (Welch’s t test). [file 40168_2016_223_MOESM13_ESM.tif]
